# Efficacy of acupuncture treatment for breast cancer-related insomnia: study protocol for a multicenter randomized controlled trial

**DOI:** 10.3389/fpsyt.2024.1301338

**Published:** 2024-05-23

**Authors:** Ping Yin, Qian Fan, Lumin Liu, Ming Yang, Shunxian Zhang, Xu Li, Wenguang Hou, Qifan Feng, Xi Wang, Zhu Jin, Fang Li, Yuelai Chen

**Affiliations:** ^1^LongHua Hospital Shanghai University of Traditional Chinese Medicine, Shanghai, China; ^2^Yueyang Hospital of Integrated Traditional Chinese and Western Medicine of Shanghai University of Traditional Chinese Medicine, Shanghai, China; ^3^Hainan Traditional Chinese Medicine Hospital, Haikou, China; ^4^Xuhui District Central Hospital of Shanghai, Shanghai, China; ^5^Shanghai Seventh People’s Hospital, Shanghai, China; ^6^Affiliated Hospital of Jiangxi University of Traditional Chinese Medicine, Nanchang, China

**Keywords:** breast cancer, insomnia, acupuncture, randomized controlled trial, protocol

## Abstract

**Background:**

Insomnia is one of the most common symptoms among breast cancer patients, which can be present throughout all stages of breast cancer. As a non-pharmacological alternative treatment, acupuncture has been suggested to improve sleep situations in patients with cancer suffering from insomnia. However, there is a lack of well-designed, high-quality clinical evidence regarding the efficacy of acupuncture in the treatment of breast cancer-related insomnia. This study is conducted to evaluate the efficacy and safety of acupuncture treatment for breast cancer-related insomnia.

**Methods:**

This study was designed as a multicenter, randomized, sham-controlled clinical trial. A total of 264 eligible patients with breast cancer-related insomnia will be randomized into an acupuncture group and a sham acupuncture group in a 1:1 ratio. In the trial, patients in the acupuncture and sham acupuncture groups will receive 12 sessions over a consecutive 4-week period. The primary outcome will be the treatment response rate of Insomnia Severity Index (ISI) at week 4; secondary outcomes include treatment remission rate of ISI, Sleep Efficiency (SE) obtained by the use of Sleep diary, treatment response rate of ISI at 8th and 16th weeks of follow-up, the mean changes of ISI, Generalized Anxiety Disorder Scale (GAD-7), Patient Health Questionnaire-9 (PHQ-9), Quality of Life Questionnaire - Core 30 (QLQ-C30), sleep parameters recorded in Actigraphy and weekly usage of remedial drugs. Adverse events will be recorded throughout the study. All analyses will be based on the ITT principle and performed with SAS 9.4 statistical software.

**Discussion:**

This trial will evaluate the clinical efficacy and safety of acupuncture for breast cancer-related insomnia. If proven effective, acupuncture will provide an effective option for patients with breast cancer-related insomnia, which will play a positive role in helping patients reduce their use of sleeping medications.

**Clinical trial registration:**

ClinicalTrials.gov, identifier NCT05510700.

## Background and objective

Insomnia is one of the most common symptoms among cancer patients. It usually refers to sleep disorders that occur among cancer patients, mainly including reduced total sleep time, difficulty in falling asleep, discontinuous sleep, easy early awakening, and inability to continue sleeping after waking up (1–5). It has been reported that about 36%-59% of cancer patients have been accompanied by insomnia at some time in their cancer trajectories (6). Insomnia may occur throughout all stages of the cancer pathways and is significantly associated with poor psychological status, physiological functioning, quality of life, increased inflammation, and possible disease progression and survival in cancer patients (7–12).

Breast cancer is one of the most common malignancies affecting women’s health, with the highest prevalence among cancers related to females (13, 14). In addition, among the various malignancies, breast cancer patients have the highest prevalence of insomnia, ranging from 42% to 69% (6), which is almost twice as high as that of the general population (6, 7, 15). Breast cancer-related insomnia, which often leads to fatigue and depression, is even associated with the risk of death (16, 17). Sick leave, unemployment, radiation, chemotherapy, and side effects of cancer treatment are all important factors contributing to insomnia in breast cancer patients (18–20). Currently, pharmacotherapy remains the most common treatment for patients with breast cancer-related insomnia (21). However, medication is only deemed beneficial for a short period (22), and long-term use will have negative effects. Although cognitive behavioral therapy (CBT) has made considerable progress in recent years (23), the use of CBT in cancer patients is complex (24) and limited. Consequently, it has become clinically crucial to figure out how to properly handle insomnia in breast cancer survivors and improve their quality of life. Fortunately, acupuncture therapy, which has a long history of use in the treatment of insomnia, has accumulated a wealth of expertise.

Acupuncture stemming from ancient theories and wisdom, has been used in the treatment of disease for thousands of years. In recent years, acupuncture has been used as a non-pharmacological intervention for the treatment of insomnia to improve clinical symptoms in patients with insomnia (25–27). It has been shown that acupuncture can increase sleep quality while addressing symptoms of mental and emotional distress (28). Additionally, acupuncture is now being used to improve patients’ sleep problems in cancer centers (29, 30). Although there have been a few clinical reports about acupuncture for cancer-caused insomnia (31–33), these studies fail to classify different types of cancer, and have small sample sizes, or the subjects come from a single center. A systematic evaluation of randomized clinical trials on acupuncture for cancer-related insomnia also has pointed out that the current level of evidence is low, that the number of clinical studies is small, and that the clinical guidance is limited, thus definite conclusions cannot be drawn (34). Therefore, high-quality clinical studies are needed to verify the clinical effectiveness of acupuncture for breast cancer-related insomnia.

In this trial, we will use a multicenter, randomized, controlled study to evaluate the clinical efficacy and safety of acupuncture in the treatment of breast cancer-related insomnia; we will make a comprehensive assessment of patients’ sleep from both subjective and objective levels. Meanwhile, we will also focus on the clinical onset time of acupuncture to observe whether acupuncture can improve patients’ sleep and alleviate breast cancer-related insomnia in short-term treatment.

This study hypothesizes that acupuncture will effectively alleviate insomnia symptoms in breast cancer patients, potentially reducing the need for sleeping medications. This study aims to provide robust evidence supporting acupuncture’s clinical benefits and informing treatment strategies for breast cancer-related insomnia.

## Methods

### Study design

**Figure 1:**
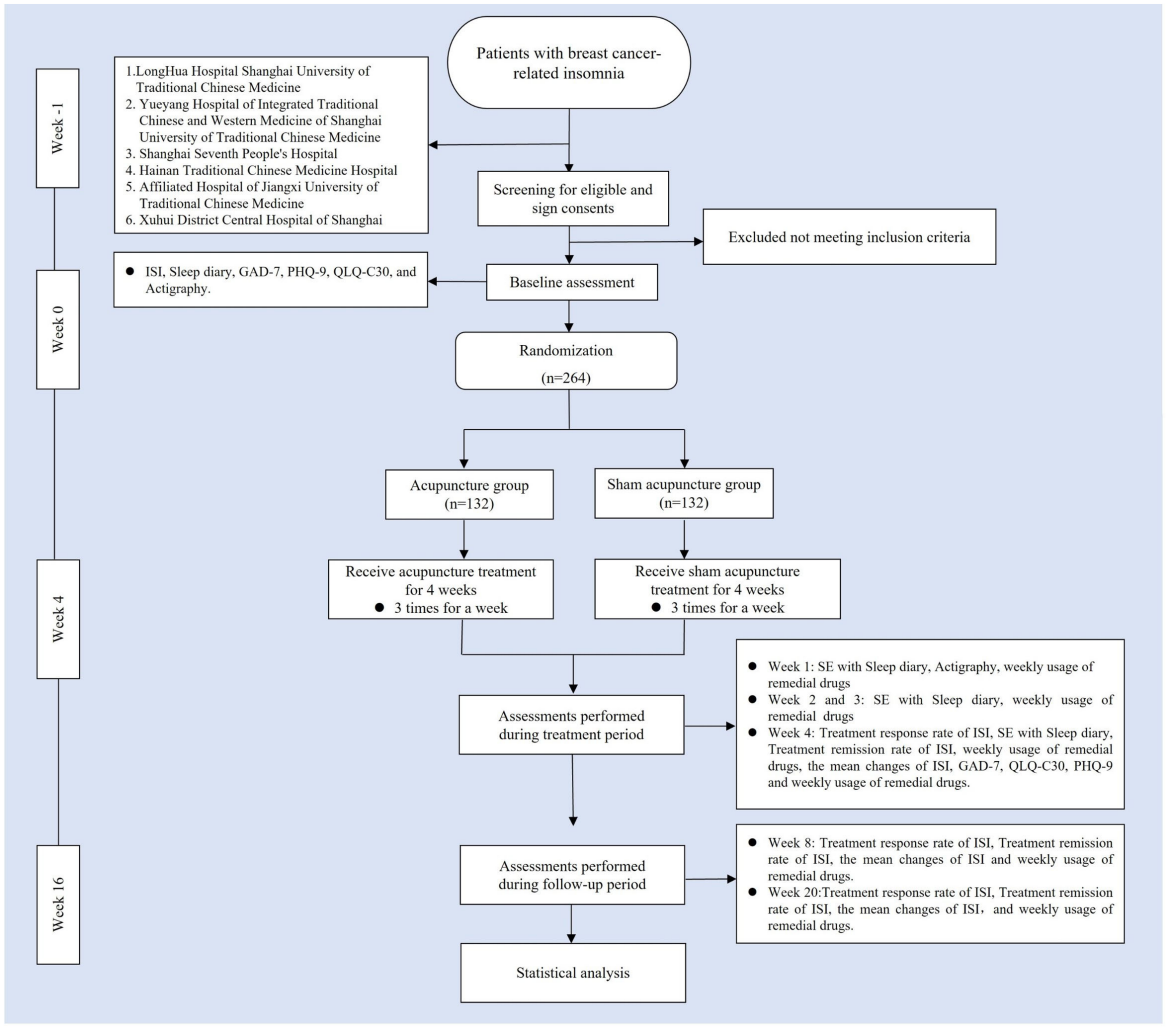
Trial flow chart. ISI, Insomia Severity Index; GAD-7, generalized Anxiety Disorder Scale; PHQ-9, Patient health Questionnaire-9; QLQ-C3O, Quality of Life Core Scale; SE, Sleep Efficiency.

This study is a multicenter, randomized, single-blind, sham acupuncture controlled trial, which will be conducted at 6 centers, including LongHua Hospital Shanghai University of Traditional Chinese Medicine, Yueyang Hospital of Integrated Traditional Chinese and Western Medicine of Shanghai University of Traditional Chinese Medicine, Shanghai Seventh People’s Hospital, Hainan Traditional Chinese Medicine Hospital, Affiliated Hospital of Jiangxi University of Traditional Chinese Medicine, and Xuhui District Central Hospital of Shanghai. The study has been approved by the Medical Ethics Committee of LongHua Hospital Shanghai University of Traditional Chinese Medicine (No. 2022LCSY032) and registered at ClinicalTrials Registry (NCT05510700). We will recruit 264 patients who meet the inclusion criteria and assign them to the acupuncture and sham acupuncture groups in a 1:1 ratio. The treatment will last 4 weeks in both groups, followed by a 12-week follow-up. A flow chart of the trial procedures is illustrated in **Figure 1** and **Table 1** with a detailed description of the study procedures and data collection.

**Table 1:** Schedule of enrolment, interventions and assessments.

Study period	Enrolment		Intervention period				Follow-up period	
Time point	Week -1	Week 0	Week 1	Week 2	Week 3	Week 4	Week 8	Week 16
Eligibility screening	**×**							
Sign informed consent	**×**							
Medical history	**×**							
Randomization		**×**						
**Intervention**								
Acupuncture			**×**	**×**	**×**	**×**		
Sham acupuncture			**×**	**×**	**×**	**×**		
**Primary outcomes**								
Treatment response rate						**×**		
**Secondary outcomes**								
Treatment remission rate						**×**	**×**	**×**
Treatment response rate							**×**	**×**
The mean changes of ISI						**×**	**×**	**×**
SE with Sleep diary		**×**	**×**	**×**	**×**	**×**		
GAD-7		**×**				**×**		
PHQ-9		**×**				**×**		
QLQ-C30		**×**				**×**		
Actigraphy sleep assessment		**×**	**×**			**×**		
Adverse events			**×**	**×**	**×**	**×**		
Weekly usage of remedial drugs			**×**	**×**	**×**	**×**	**×**	**×**
**Success of blinding**						**×**		

### Patient recruitment

All participants for this project will come from the six research centers mentioned above. Participant recruitment will be conducted through posting announcements and posters on the official web-based information platforms. Interested participants can contact researchers for initial screening through the phone numbers provided in the recruitment. Participants who initially meet the inclusion criteria will then be interviewed face-to-face and assessed at baseline with an independent researcher. After being screened by the investigators according to inclusion and exclusion criteria, subjects who meet the inclusion criteria will be informed that they will be randomly assigned to the acupuncture group or the sham acupuncture group. In addition, they will be informed of the possible benefits and risks of the study. Before entering the study, they will voluntarily sign a written informed consent, and they are free to withdraw at any time during the study.

### Inclusion criteria

The eligibility criteria that participants meet for the trial are as follows (1): Meet the diagnostic criteria for breast cancer in the 2022 2^nd^ edition of the National Comprehensive Cancer Network (NCCN) Clinical Practice Guidelines in Oncology and the American Joint Committee on Cancer (AJCC) Cancer Staging Manual (8th edition) for breast cancer staging criteria, meet the diagnostic criteria for breast cancer with TNM stage I to III; meet the diagnostic criteria for insomnia disorder within the Diagnostic and Statistical Manual of Mental Disorders, 5th Edition (DSM-5) (35): Insomnia occurred on a minimum of three nights per week and persisted for at least one month, and was related to the cancer itself or cancer-related treatment happened after breast cancer diagnosis (2). female patients aged 18–75 years (3). Eastern Cooperative Oncology Group (ECOG) performance status ≤ 2 (range, 0–5, with higher scores indicating a poorer physical condition) (4). ISI ≥ 8 (range, 0–28, with higher scores suggesting more severe insomnia) (36). (5) Predicted survival of ≥ 6 months. (6) Patients had never received acupuncture treatment. (7) No mental or intellectual abnormalities, able to understand the provisions of the scales and complete the assessment. (8) Consent to participate in this study and sign a written informed consent.

### Exclusion criteria

Patients who meet any of the following criteria are excluded from treatment: (1) Combination of more serious heart, liver, kidney, and other major diseases. (2) Patients who are pregnant or breastfeeding. (3) Those with planned surgery during the trial. (4) Previous history of drug abuse or addiction. (5) Those who have taken sedative-hypnotic drugs or antipsychotic drugs 2 weeks before the baseline visit, or received other treatment for insomnia (e.g., cognitive behavioral therapy, etc.) 3 months before the baseline visit, which may affect the efficacy of the observation. (6) Insomnia due to cancer pain with a numeric rating scale (NRS) score ≥ 4, or insomnia due to other physical diseases. (7) Long-term night work or irregular relaxation. (8) Ulcers, abscesses, skin infections, etc. at the site of needling. (9) Participation in other clinical medical trial studies within the last month.

### Randomization, allocation concealment and blinding

The random assignment scheme for this study will be generated by the Clinical Research Center of LongHua Hospital Shanghai University of Traditional Chinese Medicine with the use of the “Proc plan” program of SAS 9.4 statistical analysis software. The central randomization scheme will utilize stratified block randomization with centers as a stratification factor, competing for group inclusion. Centers notify the data management center when blocks are depleted for replenishment. Treatment assignment codes will be placed in sequentially numbered opaque envelopes by independent investigators. The envelopes will be selected by the staff members according to the order of the patients’ visits. All participants will be informed that they have an equal chance of being assigned to the acupuncture or sham acupuncture groups. Participants and researchers will not be able to anticipate groupings. In addition, to ensure shielded implementation, all investigators will receive training in the normative implementation of this study several times before the experiment is implemented, with strict adherence to the departmental separation principle.

The single-blind method is used in this trial. Except for the acupuncturist, all participants, outcome assessors, data analysts, and statisticians will be blinded during the study. We will strictly demand that communication among subjects be limited during treatment. In addition, we will schedule appointments. For the blind method to be successful, patients must wear eye masks during treatment and it is performed in a closed treatment unit. Moreover, all participants will be asked to guess which treatment they received after their last treatment to assess the success of the blinding method.

### Intervention

Participants in both groups will be given sleep hygiene instructions (e.g., be careful to avoid coffee, tea, alcohol, etc., maintain a routine, and appropriate sleep environment, and also pay attention to emotional self-regulation, etc.) through conversation, and will use it as the basic treatment of breast cancer-related insomnia. The conversations will be conducted at baseline, weeks 1, 2, 3 and 4. Meanwhile, participants will be instructed to keep a sleep diary for 5 weeks - weeks 0, 1, 2, 3 and 4. All participants will receive three treatments per week (every other day), each lasting for 30 minutes, which amounts to 12 sessions over the 4-week course. Follow-up time is week 4 and week 12 after treatment (ie, week 8 and week 16 of the whole trial period). These treatments will be administered following skin sterilization. The patients will be asked to remain supine and wear an eye mask throughout the treatment.

If patients have severe physical discomfort caused by insomnia for 2 or more consecutive days, they can take medication for sleep disorders according to their conditions, no matter what type, brand and dose, but they are required to strictly record the name, dose, and frequency of medication. Meanwhile, the investigators will meticulously document the patient’s medicine on a case report form (CRF).

### Acupuncture group

**Figure 2:**
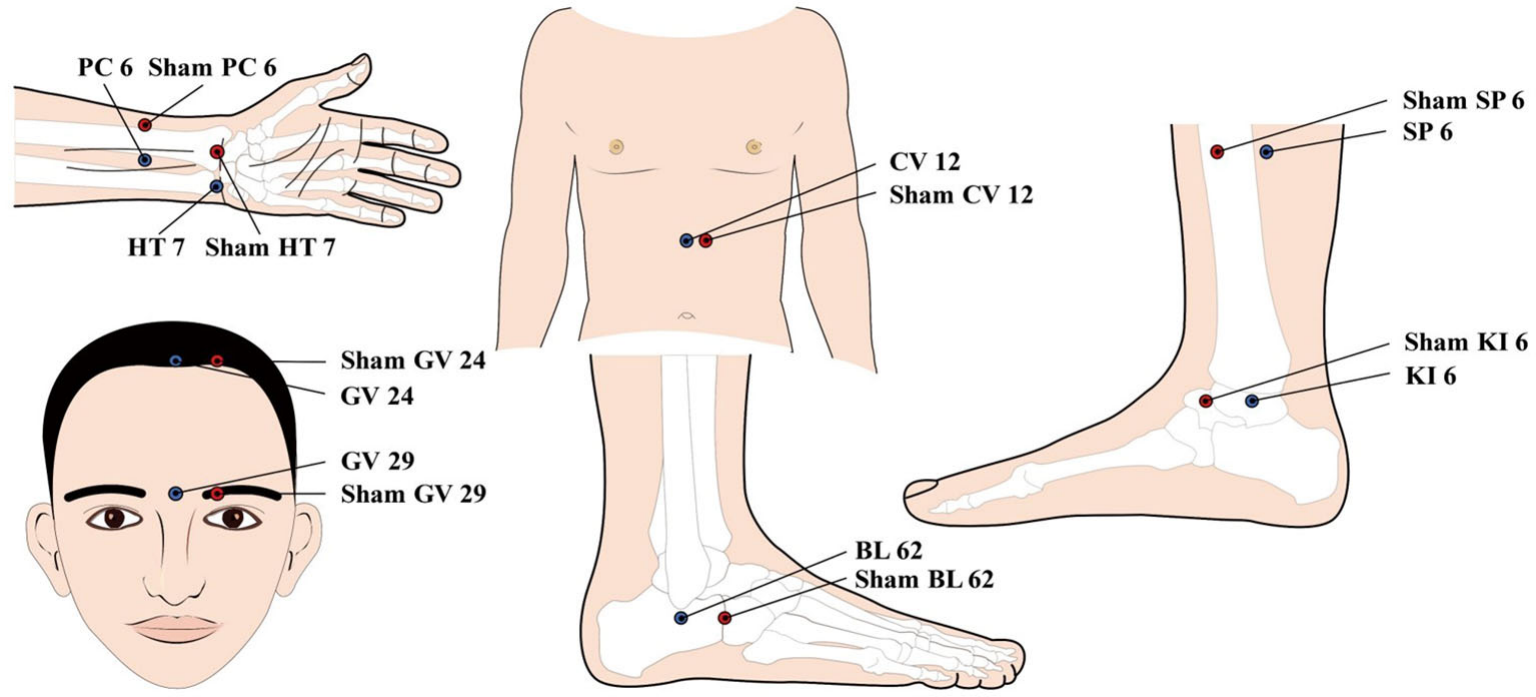
location of Acupoints for the acupuncture and Sham acupuncture Groups. PC 6; Nei Guan; HT 7: Shen Men; GV 24: Shen Ting; CV 12; Zhong Wan; SP6: san Yin Jiao; K16: Zhao Hai; BL 62: Shen Mai.

Participants in the treatment group will receive acupuncture treatment at Nei Guan (PC 6), Shen Men (HT 7), Shen Ting (GV 24), Yin Tang (GV 29), Zhong Wan (CV 12), San Yin Jiao (SP 6), Zhao Hai (KI 6), and Shen Mai (BL 62). Among the above acupoints, Nei Guan (PC 6), Shen Men (HT 7), San Yin Jiao (SP 6), Zhao Hai (KI 6), and Shen Mai (BL 62) are applied bilaterally while the rest are applied on the anterior midline (**Figure 2**). (Acupoint Location: refer to *The Location of Acupoints: State Standard of the People’s Republic of China* [GB/T 12346–2021]). The acupuncture technique for each point is described in **Table 2**. As acupuncture needles are inserted, all needles will be lifting, twirling, and thrusting to reach *de qi*, a sensation generally associated with acupuncture, including swelling, soreness, numbness, and heaviness, which is considered as an important factor in acupuncture’s therapeutic effect (37). In the study, sterile acupuncture needles will be used, which are from Suzhou Medical Supplies Factory Co.’s Huatuo (size 0.25*25mm and 0.25*40mm).

**Table 2:** The locations and manipulations of acupoints in the intervention group.

Acupoint	Location	Manipulation
Nei Guan (PC 6)	On the anterior side of the forearm, 2 *cun* above the transverse stripe of the distal palmar aspect of the wrist, between the palmaris longus tendon and the radial carpal flexor tendon.	Insert the needle perpendicularly for 0.5–1.0 *cun*.
Shen Men (HT 7)	On the anteromedial side of the wrist, the ulnar end of the palmar wrist crease, and the radial border of the flexor carpi ulnaris tendon.	Insert the needle perpendicularly for 0.3–0.5 *cun*.
Shen Ting (GV 24)	On the head, 0.5 *cun* straight above the middle of the front hairline.	Insert the needle transversely for 0.5–0.8 *cun*.
Yin Tang (GV29)	At the midpoint between the medical ends of the eyebrows.	Insert the needle transversely for 0.3–0.5 *cun*.
Zhong Wan (CV 12)	On the upper abdomen, 4 *cun* above the umbilicus, on the anterior midline.	Insert the needle perpendicularly for 1.0–1.5 *cun*.
Zhao Hai (KI 6)	On the medial side of the foot, 1 *cun* below the tip of the medial malleolus, in the depression at the lower edge of the medial malleolus.	Insert the needle perpendicularly for 0.5–0.8 *cun*.
San Yin Jiao (SP 6)	On the inner side of the calf, 3 *cun* above the tip of the medial malleolus, on the posterior border of the medial tibia.	Insert the needle perpendicularly for 1.0–1.5 *cun*.
Shen Mai (BL 62).	On the lateral side of the foot, directly below the tip of the lateral malleolus, in the depression between the lower edge of the lateral malleolus and the calcaneus.	Insert the needle perpendicularly for 0.3–0.5 *cun*.

### Sham acupuncture group

To match the true acupuncture points, sham points 1 *cun* lateral to Nei Guan (PC 6), Shen Men (HT 7), Shen Ting (GV 24), Yin Tang (GV 29), Zhong Wan (CV 12), San Yin Jiao (SP 6), Zhao Hai (KI 6), and Shen Mai (BL 62) will be used. As with the acupuncture group, sham points of Nei Guan (PC 6), Shen Men (HT 7), San Yin Jiao (SP 6), Zhao Hai (KI 6), and Shen Mai (BL 62) are applied bilaterally while the rest are applied unilaterally 1 *cun* left to the anterior midline (**Figure 2**). The placebo needles selected for this study are blunt needles without a sharp tip that cannot be pierced into the skin. The placebo needles are from Guizhou Andi Medical Equipment Co., with a size of 0.25*25mm. Meanwhile, an external patch device will hold the needles in place and these needles visually appear to be pierced into the skin. This manipulation of the placebo needles has been validated as feasible in previous clinical studies (38). At the end of the treatment, the acupuncturist will press the acupuncture point with a dry cotton ball to allow the patient to feel the “needles” being pulled out. It is worth pointing out that the sham acupuncture device used in this study has been patented by China National Intellectual Property Administration (Patent Number: ZL 2023 2 0605629.3).

## Outcomes

### Primary outcome

The Primary outcome is the treatment response rate (39, 40), which is the percentage of people whose ISI score decreased by ≥8 points at the end of week 4 treatment compared to the baseline score.

As a subjective and validated measure, the Insomnia Severity Index (ISI) assesses various aspects of sleep, including the onset of sleep, maintenance, early awakening, dissatisfaction, interference with daytime functioning, life quality, and mental distress. The scores are 0 to 4, with a total score range from 0 to 28. Higher scores indicate more severe insomnia. ISI levels can be classified as absence of insomnia (0 to 7), sub-threshold insomnia (8 to 14), moderate insomnia (15 to 21), and severe insomnia (22 to 28).

### Secondary outcome

The secondary outcomes include the following eight items (1): Treatment remission rate of ISI (40): The percentage of people with a total score of <8 points in total ISI score compared to the baseline score. It will be evaluated at weeks 4, 8 and 16. (2) SE with Sleep Diary: SE is the percentage of time spent asleep while in bed (41). It is calculated by (TST/TIB) × 100%. Sleep diary analysis will be used to determine SE subjectively which will be assessed at weeks 0, 1, 2, 3 and 4. Sleep Onset Latency (SOL), Wake After Sleep Onset (WASO), Early Morning Awakening (EMA), and Total Time in Bed (TIB) are among the sleep metrics that may be monitored using a sleep diary (41). (3) Treatment response rate of ISI at the 8th and 16th weeks of follow-up. (4) The mean changes of ISI (40) from baseline. It will be evaluated at weeks 4, 8 and 16. (5) GAD-7 (42): It is a concise anxiety self-assessment instrument consisting of 7 items to assess patients’ anxiety over the past two weeks. It will be evaluated at weeks 0 and 4. (6) PHQ-9 (43): It is a simple and validated 9-item depression self-assessment tool that assesses patients’ depression over the past two weeks. It will be evaluated at weeks 0 and 4. (7) QLQ-C30 (44): It contains 30 entries with six dimensions. It is widely used in clinical studies to assess the life quality of patients. It will be evaluated at weeks 0 and 4. (8) Sleep parameters recorded in Actigraphy (wGT3X-BT. LLC, Pensacola, USA): It provides a non-invasive monitoring method (45) that allows objective data related to sleep quality to be collected in the subject’s relatively usual and familiar natural sleep environment. It records the contents of TST, sleep awakenings (SA), WASO, average wake duration (AA) and SE, with the detection and interpretation of movements using the device’s accelerometer. We used the default settings of ActiLife for sleep situations: a sampling rate of 30 Hz in a time window of 60s (46). It will be evaluated at weeks 0, 1 and 4. (9). Weekly usage of remedial drugs: It is the percentage of participants who used emergency drugs. It will be evaluated at weeks 1, 2, 3, 4, 8 and 16.

### Blinding assessment

The independent assessor will provide participants with three choices after they have completed their last treatment: acupuncture, sham acupuncture, and uncertainty. Their choices will then be recorded. The result will be used to assess the success of the blinded implementation.

### Safety evaluation

Acupuncture’s adverse events (AEs) include the following: (1) Adverse reactions to acupuncture such as dizziness, subcutaneous hematoma, and local infection may occur. (2) Excessive acupuncture stimulation causes exacerbations. The severity of the AEs will be evaluated. Grade 1 (mild), 2 (moderate), or 3 (severe) (severe or medically significant). The investigator will assess the degrees and causes of these AEs to determine if they are connected to acupuncture or sham acupuncture. The proportion of AEs (%) during the trial is used to calculate the incidence of AEs. Any adverse event occurring during the trial should be completed on an “Adverse Event Form” and further investigated. Additionally, the process and outcome should be recorded in detail. If any serious adverse event occurs, treatment will be terminated and a final decision will be made on whether to continue the study.

### Sample size calculation

In this study, the sample size is estimated according to the treatment response rate of subjects at week 4 as the primary outcome indicator (40, 41). Based on the results of our pre-preliminary trial, the treatment response rate at week 4 was 33.3% (5/15) in the treatment group; 6.7% (1/15) in the control group; we conservatively assumed a treatment response rate of 27% at week 4 in the treatment group and 10% at week 4 in the control group. The sample size was calculated by using the “Tests for Two Proportions (Test Version)” module of PASS15 software (NCSS, LLC. Kaysville, Utah, USA) with a one-sided alpha = 0.025, power of 90%, and a 1:1 ratio. In each group, 105 cases will be needed. Considering the lost and rejected cases of 20%, the final minimum number of subjects required for the treatment and control groups will be 132 cases each, and a total of at least 264 subjects will be included.

### Statistical methods

Data will be collected for each study subject at a series of dynamic time points by the use of standardized and structured questionnaires. And then three databases will be created for this study, which are the Full analysis set (FAS), the Per-protocol set (PPS), and the Safety set (SS). For each study group of the trial, missing values were filled using the last observation carryover method, i.e., data from the last obtained data for the missing visit to the end of the observation at all time points. Statistical analyses will be performed using SAS 9.4 software (SAS Institute Inc., Cary, North Carolina 27513, US). Continuous variables are expressed as mean ± standard deviation or median (interquartile range), and categorical variables are represented by frequency (constitutive ratio). The significance level will be set at 5% (*p* < 0.05) with a 95% confidence interval.

(1) Data analysis of primary outcome: The primary outcome in the study is the treatment response rate, which is qualitative data. The treatment response rate at week 4 will be compared between the two groups by the CMH (Cochran’s and Mantel-Haenszel statistics) test, to ensure the effects of different treatment factors on the treatment response rate.
(2) Data analysis of secondary outcome: Among the secondary indexes, treatment response rate, treatment remission rate, SE with sleep diary and actigraphy, and weekly usage of remedial drugs are all qualitative data measured repeatedly, so generalized estimating equations will be used for statistics. The mean changes of ISI, SOL/WASO/EMA/TST in sleep diary, TST/SA/WASO/AA in Actigraphy, GAD, PHQ and QLQ-C30 scale are all quantitative data, therefore mixed-effects models will be used for statistics.
(3) Data analysis of safety indicators: the incidence of adverse events and medication use are qualitative data, so the chi-square test or Fisher’s test will be used for statistics.

In the process of baseline data collection before conducting the study, we added some covariates, such as anxiety, depression, age, and treatment of breast cancer. When the whole study is completed, *post hoc* analysis will be done to compare the therapeutic effects among different subgroups. To be specific, the stratified CMH test will be used to test the therapeutic effect of acupuncture on cancerous insomnia in different subgroups of breast cancer treated with different therapeutic schedules (e.g. radiotherapy, chemotherapy).

### Quality control

All study researchers will receive intensive training in the clinical research protocol, quality control, and administration before the clinical trial. For this study, licensed acupuncturists with at least 5 years of clinical experience are required. Acupuncturists will receive standardized training in acupuncture and non-acupuncture point location, acupuncture technique criteria, and how to operate sham acupuncture equipment prior to the commencement of the study. In addition, a designated clinical supervisor will regularly review the CRF, EDC and the implementation of the study. A quality control team will also be established to conduct regular or unscheduled monitoring visits, data verification, and to discuss and resolve problems that occur during the observation period in each center.

### Data collection, management and monitoring

Basic information and clinical data for all patients in this study will be collected by designated staff members via CRF and EDC. Before the clinical trial, uniform training will be provided to personnel involved in data collection, including standardization of data collection, entry and management. A dual-input approach will be employed to ensure the correctness of the data. To avoid discrepancies, two investigators will input data independently and then validate the data. Moreover, we have established a data security control team mainly consisting of experts from mainland China, such as statisticians and acupuncturists, to monitor the performance and safety of the trial. Data will be managed according to the time, content, and methods defined in the data management plan.

## Trial status

The date of the intended trial period is from October 2022 to June 2025. Patients’ recruitment started in February 2023. Following the publication of this report, the trial registration platform will be updated with any significant protocol alterations and other adjustments. The study results will be published in peer-reviewed publications or presented at academic conferences. Patients’ personal information will be anonymized prior to the publication of results to prevent the identification of individual participants.

## Discussion

With the continuous advances in medical diagnostic techniques and treatments (47), and as the increasing aging of the population (48), the survival of breast cancer patients will gradually increase and the number of long-term cancer survivors will continue to grow. Insomnia seriously affects the life quality of breast cancer patients (49). As a result, how to better settle the problem is not only the goal pursued by patients, families, and even society, but also the focus of many doctors and researchers. Currently, pharmacotherapy is more commonly used to treat patients with breast cancer-related insomnia, but there are certain potential risks. Furthermore, there is limited data to provide a sustained beneficial effect of long-term drug therapy in patients with breast cancer-related insomnia (50, 51). Yoga (52) and CBT (23, 53) have also been suggested to improve insomnia symptoms in cancer patients, but these methods often require more time from the patient (23) and have a slower onset of action (23), making their action somewhat limited. Acupuncture, a non-pharmacological therapy, that is in line with the socio-psycho-biomedical model of modern medicine, is now widely used in clinical practice for the treatment of insomnia (29–31). Although some studies focused on acupuncture for breast cancer-related insomnia (33, 54–56), there are still limitations. Previous single-arm studies initially validated acupuncture’s clinical efficacy in treating breast cancer-related insomnia (57). Recently, two randomized controlled single-blind trials compared acupuncture with placebo acupuncture to assess its efficacy. One trial focused on chemotherapy-complicated patients (33), while this study included patients at any treatment stage. This analysis could guide tailored treatment decisions. Another study assessed acupuncture’s efficacy for breast cancer-related insomnia using only the Pittsburgh Sleep Quality Index (58), while this study used multiple assessment tools for a comprehensive evaluation.

To provide a more reliable clinical basis for the acupuncture treatment of breast cancer-related insomnia, we consulted oncologists, psychiatrists, and statisticians for the study design. Consequently, we have made some improvements to the current study. Firstly, this study is designed as a multicenter randomized controlled study. In this study, six study centers are selected from three different regions of China, which will significantly reduce the risk of regional bias. Secondly, our treatment group will use a simple hand acupuncture method without combining other treatments such as electroacupuncture and auricular acupuncture. With this design, acupuncture alone can be evaluated directly for the treatment of breast cancer-related insomnia, without being confounded by other treatments. Clinically effective and commonly used acupoints are selected based on the findings of the preliminary study (59, 60). Thirdly, the control group in this study will be set up as a sham acupuncture group. Such a control group design can be used to verify the effect of needling - a real therapeutic effect or just a placebo effect and make needles less detectable to the patient. Fourthly, considering the complex psychological conditions of breast cancer patients, we won’t completely prohibit patients from taking medications for insomnia in the trial but will adopt a remedial design that allows patients to take medications according to their conditions with no restrictions of the types of medications or the doses taken if they experience serious physical discomfort due to insomnia for 2 or more consecutive days, which prevents patients from additional mental and psychological stress caused by the fear of not being able to take medication for a long period. However, we will include the patients’ medication dosage as a secondary indicator for this trial to be observed. Hence, patients will be asked to keep a detailed record, including the name of the medication, the time of administration, and the dose taken. Fifthly, our current study focuses on observing the clinical effects of 4-week acupuncture treatment and we will add a time point for assessment during the first week of treatment and use a combination of subjective scales and objective recorders to assess the short-term onset of acupuncture and the clinical efficacy at the end of treatment at week 1 and 4 of acupuncture treatment. If the therapeutic effect of acupuncture can improve the patient’s sleep within a short period, it will be crucial to build the patients’ confidence in follow-up treatment, reduce the use of medication, improve the life quality and compensate for the long-onset of other treatment methods. Finally, we will provide completely free acupuncture treatments for patients during the study period. In addition, patients will be provided with sleep hygiene instructions throughout the study. The investigators will communicate with each patient promptly, actively educate them on sleep hygiene instructions, and conduct appointment-based visits and regular follow-ups. These methods will not only reduce the burden of treating patients but also improve their compliance for the smooth implementation of the study. In conclusion, we hope that the results of this study will provide high-quality evidence for the clinical application of acupuncture in the treatment of breast cancer-related insomnia.

Nevertheless, this study still has several limitations. First of all, the specialty of the acupuncture operation makes it impossible for the acupuncturist to adopt a blinding method. Secondly, in this study, we performed clinical observation of acupuncture treatment for only 4 weeks, which is a short observation period, so the long-term efficacy of acupuncture could not be assessed. Thirdly, in this study, we performed follow-up only 4 weeks and 12 weeks after treatment, so we failed to perform long-term efficacy observation. Finally, another limitation of this study is that the point selections are all fixed. In some previous clinical studies (30, 61), the acupuncture group was selected on an individualized basis using semi-individualized, reflecting the strict adherence to TCM norms of evidence-based treatment for the specific symptoms of the participants. However, these shortcomings will be further improved in future clinical trials.

## Conclusion

In summary, it is necessary to conduct a high-quality clinical study of acupuncture for breast cancer-related insomnia. In this study, the clinical efficacy and safety of acupuncture for breast cancer-related insomnia will be evaluated. For our research, we will follow the Comprehensive Standards for Reporting Trial Reporting Guideline (62) as well as the Recommendations for Reporting Standards for Interventions in Acupuncture Clinical Trials (63). We hope that the results of this study will provide reliable clinical evidence for the treatment of breast cancer-related insomnia with acupuncture.

## Ethics statement

The trial has been approved by the Medical Ethics Committee of LongHua Hospital Shanghai University of Traditional Chinese Medicine (Ethical approval number: 2022LCSY032). All methods were carried out in accordance with relevant guidelines and regulations. Written informed consent will be required from all participants.

## Author contributions

PY: Conceptualization, Supervision, Writing – original draft. QF: Data curation, Writing – review & editing. LL: Data curation, Writing – original draft. MY: Methodology, Supervision, Writing – review & editing. SZ: Formal analysis, Software, Writing – review & editing. XL: Data curation, Supervision, Writing – original draft. WH: Project administration, Writing – review & editing. QF: Data curation, Writing – review & editing. XW: Project administration, Writing – review & editing. ZJ: Project administration, Writing – review & editing. FL: Project administration, Writing – review & editing. YC: Conceptualization, Supervision, Writing – review & editing.
